# Reversible skin microvascular hyporeactivity in patients with immune-mediated thrombocytopenic thrombotic purpura

**DOI:** 10.1186/s13054-023-04405-w

**Published:** 2023-03-21

**Authors:** Jérémie Joffre, Lisa Raia, Tomas Urbina, Vincent Bonny, Paul Gabarre, Louai Missri, Jean-Luc Baudel, Paul Coppo, Bertrand Guidet, Eric Maury, Hafid Ait-Oufella

**Affiliations:** 1 Intensive Care Unit, Saint-Antoine University Hospital, APHP, Sorbonne University, 75012 Paris, France; 2 Centre de Recherche Saint-Antoine Inserm UMR-S 938, Sorbonne University, 75012 Paris, France; 3 Hematology Department, AP-HP, Saint Antoine University Hospital, APHP, Sorbonne University, 75012 Paris, France; 4 French Reference Center for Thrombotic Microangiopathies (CNR-MAT), Saint Antoine University Hospital, APHP, Sorbonne University, 75012 Paris, France; 5 Paris Cardiovascular Research Center, Inserm U970, University Paris Cité, Paris, France

## Abstract

**Background:**

Immune-mediated thrombotic thrombocytopenic purpura (iTTP) is a rare disease characterized by arteriolar and capillary microthrombosis precipitating organ failure. However, the contribution of endothelial dysfunction on impaired microvascular blood flow in iTTP patients has been poorly explored. This pilot observational study aimed to explore endothelial-mediated vasoreactivity in iTTP patients at admission and its changes after plasma exchange therapy (PE).

**Methods:**

We conducted a prospective observational study in patients (> 18-year old) admitted in ICU for iTTP. Using laser Doppler flowmetry and acetylcholine (Ach) iontophoresis in the forearm, we recorded the skin microvascular blood flow and the endothelium-mediated vasoreactivity at admission and after PE. Demographics, biological, clinical courses, and outcomes were also collected. As a control group, we used a previously published cohort of young diabetic patients after correction of ketoacidosis.

**Results:**

Eighteen confirmed iTTP patients and 34 controls were included in the study, mainly female (72%) aged 43 ± 16-year-old. At admission, 55% had neurological abnormalities, 50% cardiac issues and 27.8% an acute kidney injury. Median platelet count was 19 G/mL [10–37]. Baseline microvascular blood flow was decreased in iTTP patients when compared to controls (5.97 ± 4.5 vs. 10.1 ± 6.3 PU, *P* = 0.03), associated with markedly impaired endothelial-mediated skin microvascular reactivity (AUC: 9627 ± 8122 vs. 16,475 ± 11,738, *P* = 0.03). Microvascular reactivity improved after the first PE session (AUC: 9627 ± 8122 vs 16,558 ± 10,699, *P* = 0.007, respectively, baseline and post-PE1) and much more after the second session (26,431 ± 23,181, *P* = 0.04 post-PE1 vs post-PE2). Hemolysis biomarkers (LDH and bilirubin) negatively correlated with skin microvascular flow and vasoreactivity.

**Conclusion:**

We highlighted a marked yet reversible skin endothelium-mediated microvascular hyporeactivity in iTTP patients that could participate in organ injury pathophysiology.

**Supplementary Information:**

The online version contains supplementary material available at 10.1186/s13054-023-04405-w.

## Introduction

Acquired or immune-mediated thrombotic thrombocytopenic purpura (TTP) is a rare thrombotic microangiopathy characterized by thrombocytopenia and hemolytic anemia [1–3]. Immune TTP is due to the presence of neutralizing anti-ADAMTS-13 autoantibodies responsible for impaired cleavage of the von Willebrand factor (VWF) mega multimers [4, 5]. Ultimately, VWF–platelet aggregates provoke microvascular thrombosis leading to inadequate microvascular blood flow, tissue ischemia and multiorgan failure. Therefore, the iTTP pattern combines hemolytic anemia, thrombocytopenia, often with neurologic, cardiac, or renal abnormalities, still associated with a 10–20% death rate [6, 7]. Current treatment consists of plasma exchange (PE) [8–10] combined with immunosuppressive therapy (e.g., glucocorticoids and rituximab) and caplacizumab [11], an anti–VWF factor monoclonal humanized antibody inhibiting interaction between VWF multimers and platelets [12, 13].

Arteriolar and capillary microthrombosis due to the accumulation of VWF–platelet aggregates lead to life-threatening organ hypoperfusion, affecting the heart and the brain. However, the consequences of VWF–platelet aggregates on the endothelium, a key regulator of blood flow, remain unknown. This observational study aimed to explore endothelial-dependent microvascular reactivity in iTTP patients in the intensive care unit (ICU) at admission and after treatment.

## Methods

We conducted a prospective observational study in our tertiary university hospital. We included iTTP patients (> 18-year old) experiencing their first acute event, admitted to our ICU between January 2016 and September 2022. Clinical and biological parameters were recorded. Skin microcirculatory reactivity in the right forearm area was recorded at ICU admission (baseline) and after PE. As a control group for microvascular reactivity, we used data from a previously published cohort of young diabetic patients recorded after the correction of metabolic acidosis [14]. The local ethical committee approved the protocol (Comité de Protection des Personnes, Hôpital Saint-Louis, France, No 2015/64NI), and the database was registered according to the French legislation (No 2,228,742), and all patients consented to anonymous data use for academic research and publication. It was a noninvasive observational study without any specific intervention. All patients were managed following international guidelines for TTP [15] and in collaboration with the physician of the thrombotic microangiopathy national reference center. All patients received urgent therapeutic PE (1.5 plasmatic mass, 100% fresh frozen plasma (FFP)), corticosteroids, and 17/18 patients received caplacizumab on the first day of ICU admission.

### Skin microcirculatory endothelial function assessment

We recorded microvascular parameters at baseline and after PE, using laser Doppler flowmetry and acetylcholine iontophoresis in the forearm area (Additional file 1: Figure S1). Methods have been previously described and validated by our group in different clinical settings (15–18). Briefly, a calibrated laser Doppler flow meter probe (Periflux 5000; Perimed, Craponne, France) embedded within a drug delivery chamber loaded with 80 μg of acetylcholine (Miochol; Novartis, Cedex, France) was used in combination with a current delivering generator. After 1 min of baseline microvascular blood flow recording, three successive current pulses (0.12 mA, 12 ms) were delivered, leading to acetylcholine diffusion within small skin vessels. Microcirculatory skin blood flow was recorded for 10 min following the first impulse. Baseline blood flow (expressed as flow index), maximal blood flow (peak value), and area under the curve (AUC) after acetylcholine iontophoresis were determined for each patient at each time point, and curves were blindly analyzed offline (a representative record is shown as Additional file 1: Fig. S1).

### Statistics

Continuous variables were presented as mean ± SD or median and 25th–75th interquartile ranges (IQR). Discrete variables were presented as percentages. Comparisons between groups were made with Fisher test for discrete variables and Mann–Whitney *U* test for continuous variables. Comparisons between admission and post-PE values were made using a paired Wilcoxon signed-rank test. Statistical analysis and graphical representations were performed using GraphPad Prism 10.2 software (Graph Pad Software Inc., La Jolla, CA). A two-sided *P*-value of less than 0.05 was considered statistically significant.

## Results

Eighteen consecutive iTTP patients were included, 72% were female, aged 43 ± 16-year-old. ADAMTS13 activity at baseline was below 10% in all included patients [16] (below 5% in 15 patients (83.3%)) and all included patients had circulating anti-ADAMTS13 autoantibodies, which confirmed the final diagnosis of iTTP. At ICU admission, 55% had neurological abnormalities, 50% cardiac (troponin elevation, EKG, or echocardiography) issues and 27.8% had stage ⩾1 acute kidney injury according to KDIGO classification [17]. At baseline, all included patients had severe thrombocytopenia with median platelet count at 19 G/mL [10–37] G/L, mild regenerative anemia (hemoglobin: 9.6 g/dl [7.6–10], reticulocytes 178 [128–285]G/L) associated with hemolysis markers (median bilirubin: 36 µmol/L [23–59], haptoglobin: 0 g/dL [0–0.035]). Only one patient was under mechanical ventilation and received vasopressors. None received renal replacement therapy. All patients were treated by PE (median number of PE: 4 [2–5]), 100% corticosteroids (methylprednisolone 1 mg/Kg/Day i.v), and 88.9% caplacizumab (one injection i.v. before the first PE, then 10 mg/Day s.c). Patients' baseline characteristics are reported in Table 1 and initial treatment and ICU stay characteristics are summarized in Additional file 1: Table S1. Overall, the in-ICU length of stay was 6.4 [4.8–8] days, and one patient died in ICU (5.5%).

**Table 1:** Baseline patients’ characteristics

Baseline patient’s characteristics	iTTP (*n* = 18)	Controls (*n* = 34)	*P* value
Age (years. Mean ± SD)	43 ± 16	44 ± 15	0.86
Male (*n*%)	5 (28)	23 (68)	0.009
Medical history (*n*%)			
Diabetes mellitus	3 (17)	34 (100)	< 0.0001
Hypertension	5 (28)	7 (21)	0.73
Cardiopathy	1 (5.6)	3 (8.8)	> 0.99
Peripheral vascular disease	1 (5.6)	1 (2.9)	> 0.99
CKD	0 (0)	7 (21)	0.08
Neurological disease	1 (5.6)	0 (0)	0.35
Cirrhosis	0 (0)	1 (2.9)	> 0.99
Vitals (Mean ± SD)			
Core temperature (°C)	37 ± 0.85	37 ± 0.34	0.41
SBP (mmHg)	135 ± 20	135 ± 25	0.87
HR (bpm)	89 ± 12	106 ± 14	0.0001
CRT (sec)	1.3 ± 1.2	1.7 ± 0.9	0.15
Mottling score	0.3 ± 0.8	0.2 ± 0.5	0.74
Biologicals (Median [IQR])			
Hemoglobin (g/dL)	9.6 [7.6–10]	13 [12–15]	< 0.0001
WBC (10^3^/mL)	10.59 [7.8–13.6]	10.51[6.5–14.4]	0.87
Platelets (10^6^/mL)	19.5 [9.75–37]	229 [145–272]	< 0.0001
Urea (mmol/L)	6.5 [5.1–9.2]	4.7 [3.1–9.5]	0.05
Creatinine (µmol/L)	73 [54–115]	76 [ 48–102]	0.99
Haptoglobin (g/L)	0 [0–0.035]	–	–
Bilirubin (µmol/L)	36 [23–59]	–	–
LDH (UI/L)	1494 [942–2423]	–	–
Schizocytes (%)	2.5 [1.5–4.4]	–	–
Troponin us (ng/L)	13 [0.52–236]	–	–
BNP (ng/L)	68 [20–155]	–	–
Lactate (mmol/L)	1.4 [0.9–2.1]	1.1 [0.8–1.9]	0.55
ADAMTS 13			
< 5%	15 (83.3)	–	–
< 10%	3 (16.7)	–	–
Ab Anti-ADAMTS13 positivity	18 (100)	–	–
SAPS II	17.2 ± 12.7	30.1 ± 18	< 0.0001

*iTTP* immune-mediated thrombocytopenic thrombotic purpura, *SD* standard deviation, *BMI* body mass index, *CKD* chronic kidney disease, *SBP* systolic blood pressure, *HR* heart rate, *IQR* interquartile range, *WBC* white blood cells, *LDH* lactate dehydrogenase, *BNP* brain natriuretic peptide, *Ab* antibodies, *SOFA* Sequential Organ Failure Assessment, *CRT* capillary refill time

**Fig. 1:**
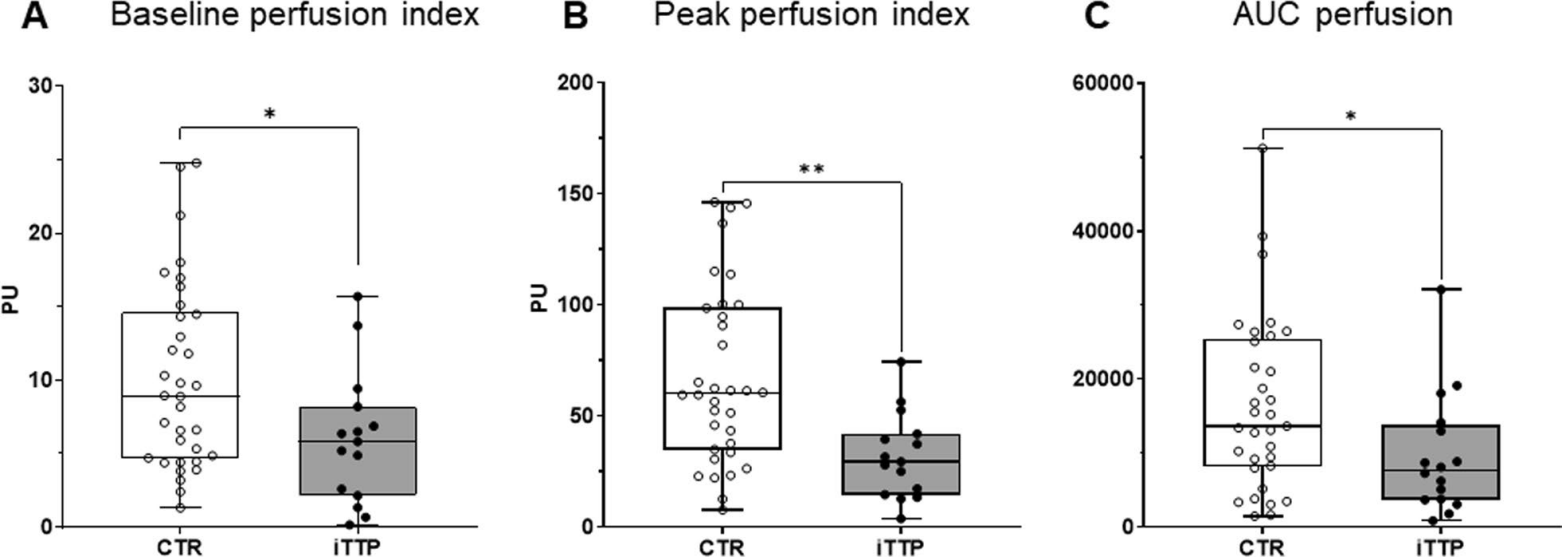
Skin microvascular endothelium-mediated reactivity assessed by laser Doppler flowmetry at baseline in iTTP and controls. Comparison of skin microvascular laser Doppler flowmetry value between controls and iTTP patients at admission regarding the baseline flow index (**A**) and the response to Ach iontophoresis (Peak value (**B**) and AUC (**C**)). *P < 0.05, **P < 0.01, CTR versus ITTP, two-tailed Mann–Whitney *U* test. **B** Abbreviations: PU, perfusion index; CTR, controls, iTTP, immune-mediated thrombocytopenic thrombotic purpura; AUC, area under curve

**Fig. 2:**
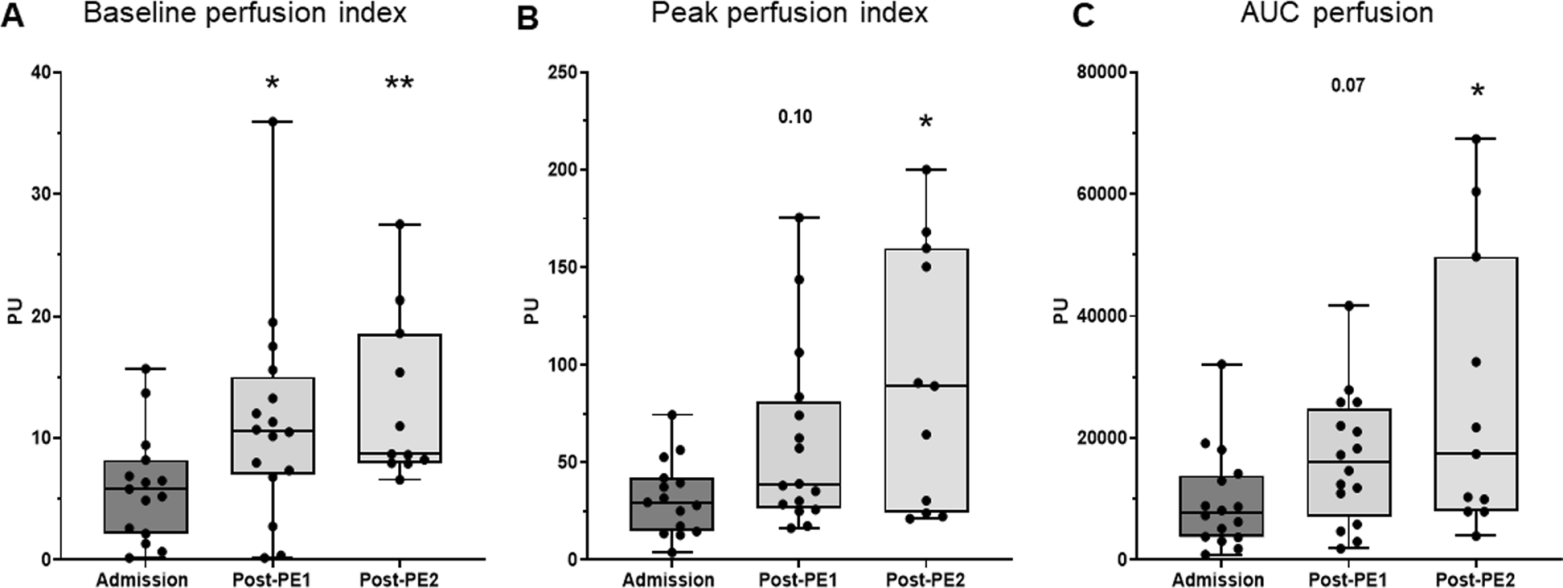
iTTP patients’ skin microvascular endothelium-mediated reactivity assessed by laser Doppler flowmetry at baseline and after PE. Evolution of skin microvascular laser Doppler flowmetry value regarding the baseline flow index (**A**) and the response to Ach iontophoresis (Peak value (**B**) and AUC (**C**)), in iTTP patients at admission and after the two first PE. *P < 0.05, **P < 0.01, paired Wilcoxon signed-rank test at each time point versus admission value for. Abbreviations: PU, perfusion index; CTR, controls, iTTP, immune-mediated thrombocytopenic thrombotic purpura; PE, plasma exchange; AUC, area under curve; Ach, Acetylcholine

First, we compared the microvascular reactivity of iTTP patients with a cohort of diabetic patients admitted to our ICU after correction of keto-acidosis. Such a control cohort was relevant because patients were young with rare co-morbidities (Table 1) and no severe organ failure. At admission, when compared to the control group (Fig. 1 and Additional file 1: Table S2), we observed that iTTP patients had twofold lower skin microvascular blood flow (5.97 ± 4.5 vs. 10.1 ± 6.3 PU, *P* = 0.03) (Fig. 1A). In addition, we found marked impaired endothelial-mediated microvascular reactivity in iTTP patients characterized by a lower peak after Ach iontophoresis (31.9 ± 19.1 vs. 67.7 ± 39.9, *P* = 0.001) (Fig. 1B) and ultimately a lower AUC (9627 ± 8122 vs. 16,475 ± 11,738, *P* = 0.03) (Fig. 1C) (Fig. 2).

**Fig. 3:**
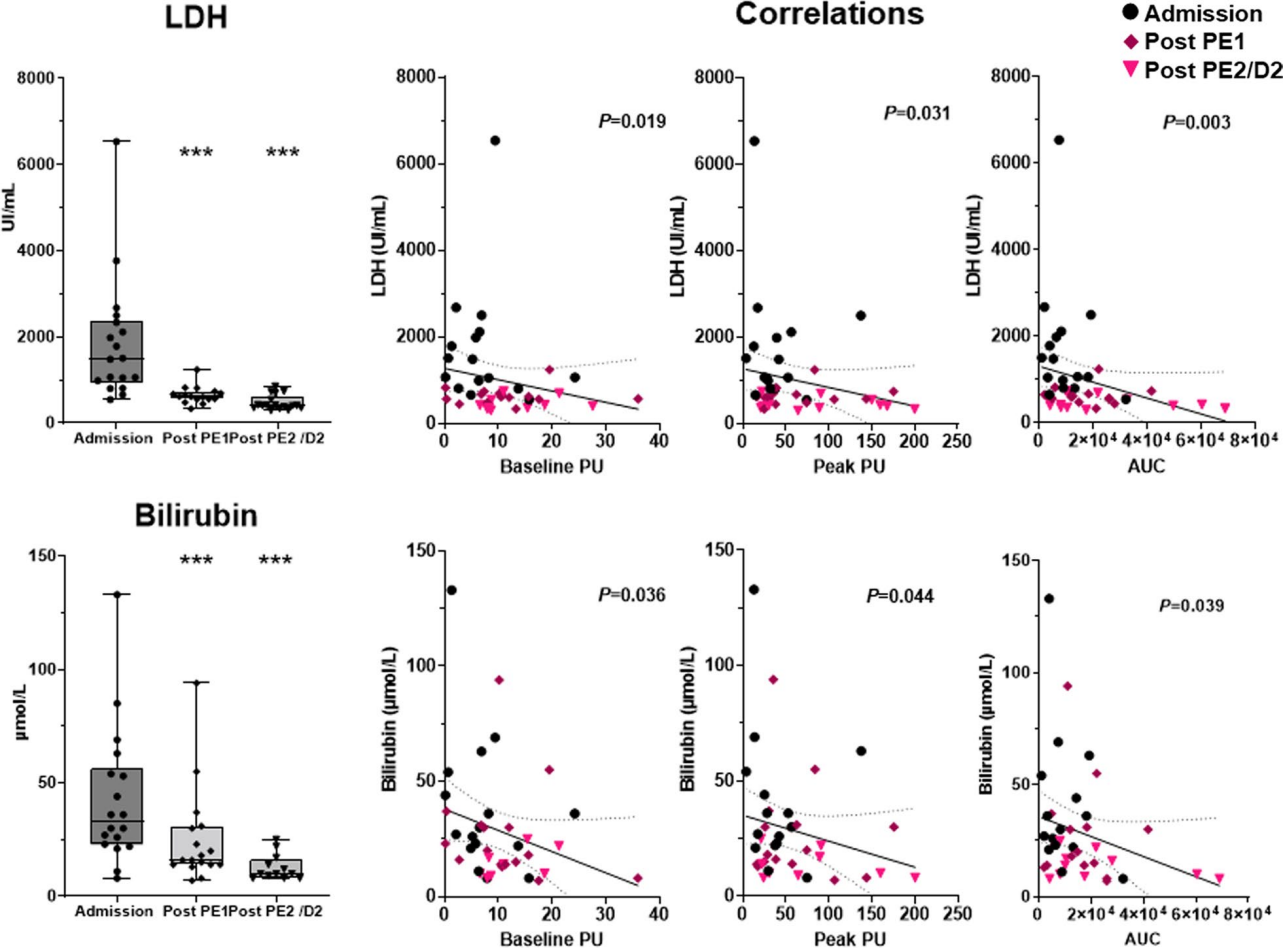
Biological variations during ICU stay and correlation with microvascular perfusion parameters. Courses of biomarkers in iTTP patients during the first days in ICU and correlation with flowmetry values. Hemolysis biomarkers (LDH and bilirubin) negatively correlate with microvascular flow and reactivity ***P < 0.0001, versus admission value, paired Wilcoxon signed-rank test at each time point. On correlations graph, the full line represents the linear regression and the dotted line show the 95%IC. Abbreviations: iTTP, immune-mediated thrombocytopenic thrombotic purpura; PE, plasma exchange; LDH, lactate dehydrogenase; PU, perfusion unit

**Fig. 4:**
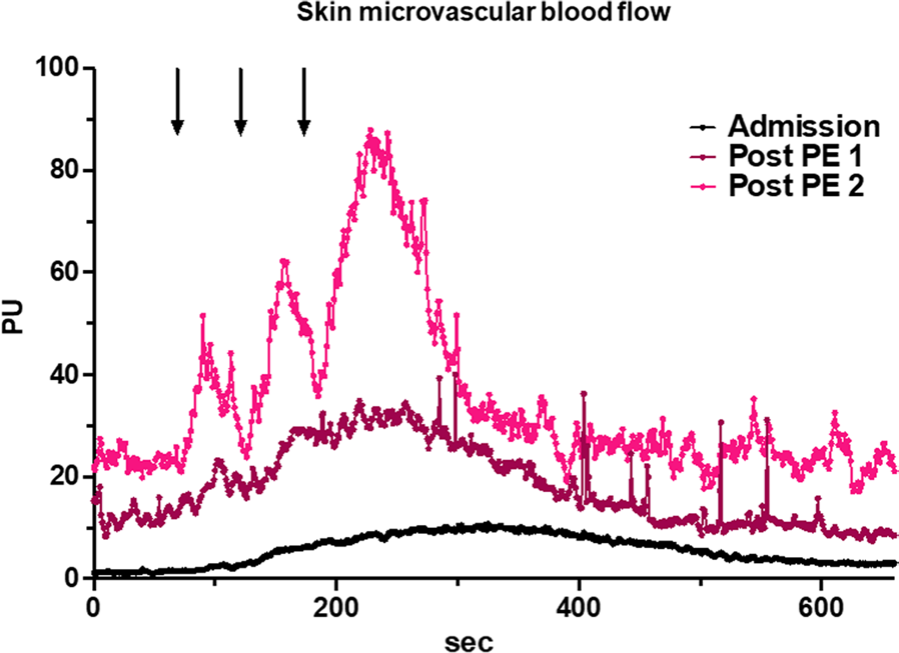
Archetypical record of the gradual improvement of skin microvascular reactivity following PE. Example of the gradual improvement of the skin microvascular reactivity in a single iTTP patient. Arrows indicate the successive Ach iontophoresis application. Abbreviations: PU, perfusion index; iTTP, immune-mediated thrombocytopenic thrombotic purpura; PE, plasma exchange; Ach, Acetylcholine

Next, on iTTP patients, we analyzed the impact of combined treatment on endothelial-dependent microvascular hyporeactivity during ICU stay. Acetylcholine iontophoresis was repeated after the first and the second PE session. After the first PE session, platelet count significantly increased (26 ± 28 *vs* 42 ± 38 G/mL, *P* = 0.0003) while hemolysis parameters improved (LDH 1870 ± 1440 vs. 650 ± 203 UI/mL, *P* < 0.0001, Haptoglobin 0.09 ± 0.2 vs. 0.56 ± 0.16 g/L, *P* < 0.0001) and biological recovery was more pronounced after the second session (Additional file 1: Table S1, Fig. 3 and Additional file 1: Fig. S2A). We observed that global microvascular blood flow significantly increased after the first PE session (Baseline perfusion index: 5.97 ± 4.5 PU at admission *vs* 11.38 ± 8.6 post-PE1, *P* = 0.027,) and even more after the second session (Baseline perfusion index 12.89 ± 6.9 PU, *P* = *0.008 vs* admission). Global microvascular reactivity improved after the first PE session (AUC: 9627 ± 8122 *vs* 16,558 ± 10,699, *P* = 0.007, respectively, baseline and post-PE1) and much more after the second session (26,431 ± 23,181, *P* = 0.04 post-PE1 vs post-PE2) (Fig. 2A–C and Additional file 1: Table S2). Changes in microvascular reactivity after PE were heterogeneous, some patients improved after the first PE while others improved their microvascular reactivity after the second. Finally, few iTTP patients had no variation of skin microvascular response to Ach (Additional file 1: Fig. S3). Figure 4 shows an archetypical example of endothelium-mediated microvascular hyporeactivity in a single patient which improved after the plasma exchange session and much more after the second session. Interestingly, microvascular blood flow across time positively correlated with platelet but not the vasoreactivity (Additional file 1: Fig. S2A). Hemolysis biomarkers (LDH and bilirubin) negatively correlated with microvascular flow and reactivity (Fig. 3). Conversely, we observed no correlation between microvascular flow/ reactivity and hemoglobin, haptoglobin, schizocytes or reticulocytes (Additional file 1: Fig. S2B).

## Discussion

In this prospective study, we showed a markedly impaired skin microvascular endothelial-mediated reactivity in iTTP patients which recovered quickly after plasma exchange therapy.

This profoundly impaired microvascular vasoreactivity is similar to what our group previously observed in other critical conditions characterized by patent endothelial dysfunction such as critically ill COVID-19 [18] septic shock [19] or severe keto-acidosis [14]. Endothelial cell (EC) involvement in the pathophysiology of thrombotic microangiopathy-associated organ failure has been suggested in animal models but remains poorly demonstrated in humans. Indeed, experimental models indicated that *Adamts-13* deficiency, by itself, is not sufficient to trigger thrombotic microangiopathy. Endothelial activation is another necessary step to induce microvascular disease, probably by releasing of a large amount of UL-VWF [20–22]. In the same line, in primates, the injection of human anti-ADAMTS-13 neutralizing autoantibody provokes a transient biological thrombotic microangiopathy but not a severe disease responsible for organ failure [23]. In iTTP patients, circulating EC number and plasma biomarkers reflecting endothelial activation are increased and correlated with the outcome supporting a role of the endothelium in the pathophysiology of iTTP [24]. Recently, Tellier et al., reported that several plasmatic components, including anti-ADAMTS13 IgG, free heme and possibly others converge to induce EC activation ex vivo [25]. Interestingly the intensity of the ex vivo EC activation induced by iTTP patients' plasmas correlated with the disease severity [25]. Among soluble factors released in iTTP, hemolysis-derived products (including cell-free heme, free-hemoglobin and bilirubin at high concentration [26, 27]) are well known to be highly toxic for the endothelium [28, 29]. Moreover, in a murine model, Frei et al. reported that hemolysis causes direct vascular injury and functionally impaired vasodilation via increased scavenging of nitric oxide by plasma free hemoglobin [30]. Interestingly, in our study, we observed a negative correlation between endothelium-mediated skin microvascular vasodilation and hemolysis parameters.

We showed that the microvascular reactivity of iTTP patients is impaired and, therefore, could participate in organ injury besides the thrombotic process. Moreover, the endothelial vasoreactivity is restored after plasma exchange therapy at the same time than platelet count recovered. However, the improvement of endothelial reactivity could not be directly linked to exchange plasma therapy because at the same time, almost all the patient received additional treatment including steroids and Caplacizumab. Currently, there is no experimental data about the effect of Caplacizumab on endothelial microvascular reactivity [31].

We acknowledge some limitations to this observational and translational study. First, this is a single-center study with a limited number of patients. Second, given the multiple treatments received simultaneously and the synchronous correction of thrombocytopenia and hemolysis, one cannot speculate on which biological mechanism is responsible for the microcirculatory improvement. Next, as a control group, we used a previously published cohort of young diabetic patients after correction of ketoacidosis where we showed that microvascular hyporeactivity recovered after acidosis correction [14] Given that patients with cardiovascular risk factors are susceptible to a lower microvascular reactivity; difference between healthy subjects and iTTP patients could be even more important than differences between diabetic and iTTP patients [32–35]. The device used in this study only allows exploration of the skin microvasculature and we did not investigate the endothelium of key organs affected by iTTP such as the brain, heart and kidney microcirculation [36, 37]. Finally, we did not explore the endothelium-independent vasodilation, which requires either heating or nitroprusside challenge. Thus, we cannot rule out that iTTP patients have impaired endothelium-independent vasodilation reserve [38] or decreased NO bioavailability [39], on top of the observed impaired endothelium-mediated vasoreactivity.

## Conclusion

This prospective observational study highlights a marked endothelium-mediated microvascular hyporeactivity in acute iTTP patients that could participate in organ injury pathophysiology. Moreover, endothelium-mediated vasoreactivity dysfunction quickly recovered after PE therapy.

## Supplementary Information

**Additional file 1. **Supplementary Figures and Tables.

## Data Availability

The datasets used and analyzed during the current study are available from the corresponding author upon reasonable request.

## References

[1] Moake JL (2002). Thrombotic microangiopathies. N Engl J Med.

[2] George JN (2006). Clinical practice: thrombotic thrombocytopenic purpura. N Engl J Med.

[3] George JN, Nester CM (2014). Syndromes of thrombotic microangiopathy. N Engl J Med.

[4] Rieger M, Mannucci PM, Kremer Hovinga JA, Herzog A, Gerstenbauer G, Konetschny C, Zimmermann K, Scharrer I, Peyvandi F, Galbusera M (2005). ADAMTS13 autoantibodies in patients with thrombotic microangiopathies and other immunomediated diseases. Blood.

[5] Thomas MR, de Groot R, Scully MA, Crawley JT (2015). Pathogenicity of anti-ADAMTS13 autoantibodies in acquired thrombotic thrombocytopenic purpura. EBioMedicine.

[6] Benhamou Y, Assie C, Boelle PY, Buffet M, Grillberger R, Malot S, Wynckel A, Presne C, Choukroun G, Poullin P (2012). Development and validation of a predictive model for death in acquired severe ADAMTS13 deficiency-associated idiopathic thrombotic thrombocytopenic purpura: the French TMA Reference Center experience. Haematologica.

[7] Peigne V, Perez P, Resche Rigon M, Mariotte E, Canet E, Mira JP, Coppo P, Veyradier A, Azoulay E (2012). Causes and risk factors of death in patients with thrombotic microangiopathies. Intensive Care Med.

[8] Adamski J (2014). Thrombotic microangiopathy and indications for therapeutic plasma exchange. Hematology Am Soc Hematol Educ Progr.

[9] Nguyen TC, Han YY (2011). Plasma exchange therapy for thrombotic microangiopathies. Organogenesis.

[10] Winters JL (2017). Plasma exchange in thrombotic microangiopathies (TMAs) other than thrombotic thrombocytopenic purpura (TTP). Hematol Am Soc Hematol Educ Progr.

[11] Scully M, Cataland SR, Peyvandi F, Coppo P, Knobl P, Kremer Hovinga JA, Metjian A, de la Rubia J, Pavenski K, Callewaert F (2019). Caplacizumab treatment for acquired thrombotic thrombocytopenic purpura. N Engl J Med.

[12] George JN (2012). Corticosteroids and rituximab as adjunctive treatments for thrombotic thrombocytopenic purpura. Am J Hematol.

[13] Sukumar S, Lammle B, Cataland SR (2021). Thrombotic Thrombocytopenic Purpura: Pathophysiology, Diagnosis, and Management. J Clin Med.

[14] Joffre J, Bourcier S, Hariri G, Miailhe AF, Bige N, Dumas G, Dubee V, Boelle PY, Abdallah I, Baudel JL (2018). Reversible microvascular hyporeactivity to acetylcholine during diabetic ketoacidosis. Crit Care Med.

[15] Zheng XL, Vesely SK, Cataland SR, Coppo P, Geldziler B, Iorio A, Matsumoto M, Mustafa RA, Pai M, Rock G (2020). ISTH guidelines for treatment of thrombotic thrombocytopenic purpura. J Thromb Haemost.

[16] Lotta LA, Wu HM, Musallam KM, Peyvandi F (2013). The emerging concept of residual ADAMTS13 activity in ADAMTS13-deficient thrombotic thrombocytopenic purpura. Blood Rev.

[17] Summary of recommendation statements. Kidney Int Suppl (2011) 2012; 2(1): 8–12. 10.1038/kisup.2012.7 PMC4089654 25018916

[18] Raia L, Urbina T, Gabarre P, Bonny V, Hariri G, Ehrminger S, Bige N, Baudel JL, Guidet B, Maury E (2022). Impaired skin microvascular endothelial reactivity in critically ill COVID-19 patients. Ann Intensive Care.

[19] Bourcier S, Joffre J, Dubee V, Preda G, Baudel JL, Bige N, Leblanc G, Levy BI, Guidet B, Maury E (2017). Marked regional endothelial dysfunction in mottled skin area in patients with severe infections. Crit Care.

[20] Joffre J, Hellman J, Ince C, Ait-Oufella H (2020). Endothelial responses in sepsis. Am J Respir Crit Care Med.

[21] Raia L, Zafrani L (2022). Endothelial activation and microcirculatory disorders in sepsis. Front Med.

[22] Motto DG, Chauhan AK, Zhu G, Homeister J, Lamb CB, Desch KC, Zhang W, Tsai HM, Wagner DD, Ginsburg D (2005). Shigatoxin triggers thrombotic thrombocytopenic purpura in genetically susceptible ADAMTS13-deficient mice. J Clin Invest.

[23] Feys HB, Roodt J, Vandeputte N, Pareyn I, Lamprecht S, van Rensburg WJ, Anderson PJ, Budde U, Louw VJ, Badenhorst PN (2010). Thrombotic thrombocytopenic purpura directly linked with ADAMTS13 inhibition in the baboon (*Papio ursinus*). Blood.

[24] Widemann A, Pasero C, Arnaud L, Poullin P, Loundou AD, Choukroun G, Sanderson F, Lacroix R, Sabatier F, Coppo P (2014). Circulating endothelial cells and progenitors as prognostic factors during autoimmune thrombotic thrombocytopenic purpura: results of a prospective multicenter French study. J Thromb Haemost.

[25] Tellier E, Widemann A, Cauchois R, Faccini J, Lagarde M, Brun M, Robert P, Robert S, Bachelier R, Poullin P (2022). Immune thrombotic thrombocytopenic purpura plasmas induce calcium- and IgG-dependent endothelial activation: correlations with disease severity. Haematologica.

[26] Maruhashi T, Kihara Y, Higashi Y (2019). Bilirubin and endothelial function. J Atheroscler Thromb.

[27] Brito MA, Palmela I, Cardoso FL, Sa-Pereira I, Brites D (2014). Blood-brain barrier and bilirubin: clinical aspects and experimental data. Arch Med Res.

[28] Frimat M, Boudhabhay I, Roumenina LT (2019). Hemolysis derived products toxicity and endothelium: model of the second hit. Toxins.

[29] Aslan M, Ryan TM, Adler B, Townes TM, Parks DA, Thompson JA, Tousson A, Gladwin MT, Patel RP, Tarpey MM (2001). Oxygen radical inhibition of nitric oxide-dependent vascular function in sickle cell disease. Proc Natl Acad Sci USA.

[30] Frei AC, Guo Y, Jones DW, Pritchard KA, Fagan KA, Hogg N, Wandersee NJ (2008). Vascular dysfunction in a murine model of severe hemolysis. Blood.

[31] Goshua G, Sinha P, Hendrickson JE, Tormey C, Bendapudi PK, Lee AI (2021). Cost effectiveness of caplacizumab in acquired thrombotic thrombocytopenic purpura. Blood.

[32] Debbabi H, Bonnin P, Ducluzeau PH, Leftheriotis G, Levy BI (2010). Noninvasive assessment of endothelial function in the skin microcirculation. Am J Hypertens.

[33] Kihara M, Low PA (1995). Impaired vasoreactivity to nitric oxide in experimental diabetic neuropathy. Exp Neurol.

[34] Debbabi H, Bonnin P, Levy BI (2010). Effects of blood pressure control with perindopril/indapamide on the microcirculation in hypertensive patients. Am J Hypertens.

[35] Vuletic V, Cengic L, Basic S, Sporis D, Rahelic D, Demarin V (2011). Impaired cerebral vasoreactivity in type 2 diabetes mellitus. Coll Antropol.

[36] Gomez-Segui I, Pascual Izquierdo C, Mingot Castellano ME, de la Rubia CJ (2023). An update on the pathogenesis and diagnosis of thrombotic thrombocytopenic purpura. Expert Rev Hematol.

[37] Fodil S, Zafrani L (2022). Severe thrombotic thrombocytopenic purpura (TTP) with organ failure in critically Ill patients. J Clin Med.

[38] Alba BK, Greaney JL, Ferguson SB, Alexander LM (2018). Endothelial function is impaired in the cutaneous microcirculation of adults with psoriasis through reductions in nitric oxide-dependent vasodilation. Am J Physiol Heart Circ Physiol.

[39] Greaney JL, Saunders EFH, Santhanam L, Alexander LM (2019). Oxidative stress contributes to microvascular endothelial dysfunction in men and women with major depressive disorder. Circ Res.

