# Supplementary material for: Reversible skin microvascular hyporeactivity in patients with immune-mediated thrombocytopenic thrombotic purpura
Source: Crit Care. 2023 Mar 21;27:116. doi: 10.1186/s13054-023-04405-w (PMC10028781; doi:10.1186/s13054-023-04405-w)
Supplement: Supplementary file 1 — Additional file 1. Supplementary Figures and Tables. [file 13054_2023_4405_MOESM1_ESM.docx]

# **Supplemental materials**

**Supplemental Fig. 1: Method to analyze the flowmetry and iontophoresis data offline**


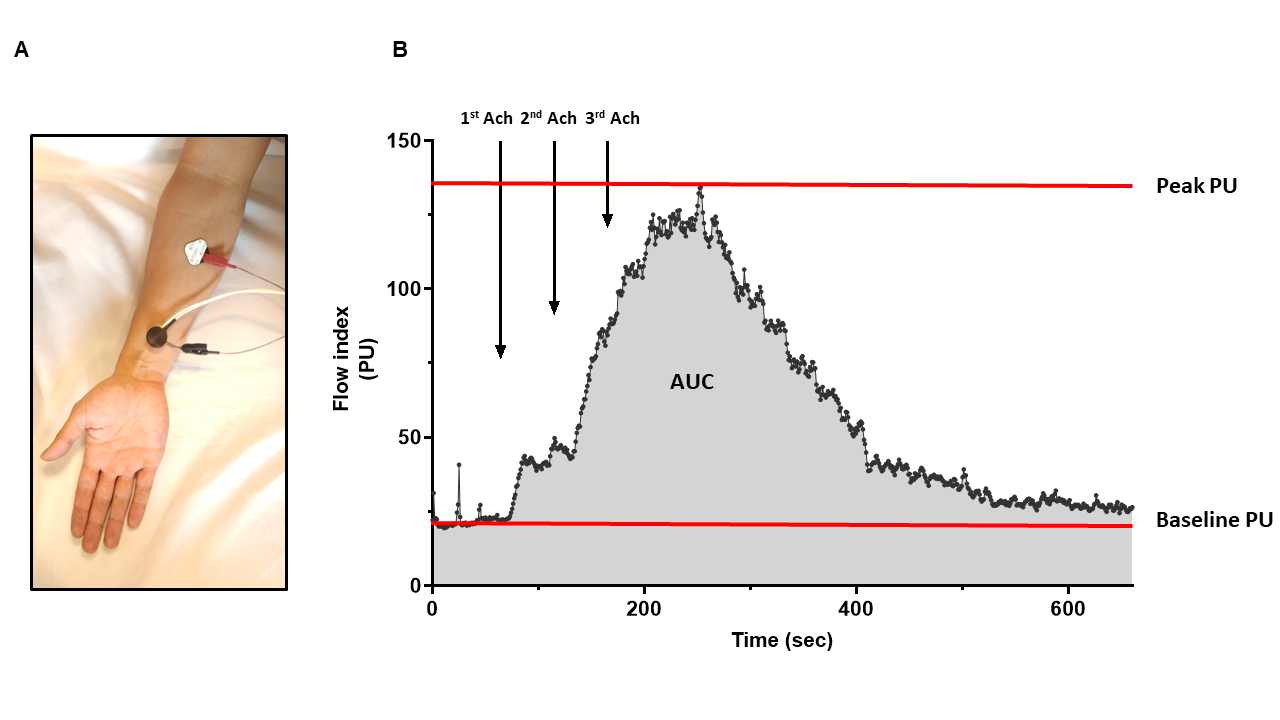


**Supplemental Figure 1 Legend. (A)** shows the forearm area where the iontophoresis was applied and the skin microvascular flow recorded. **(B)** shows a typical recording of microvascular skin blood flow recorded by laser doppler flowmetry baseline and following 3 successive iontophoretic applications of Acetylcholine (Healthy volunteer). Abbreviations: AUC, area under curve; Ach, Acetylcholine.

**Supplemental table 1: iTTP initial presentation, treatments and ICU stay characteristics**

| **iTTP initial treatment and ICU stay characteristics** | **(n=18)** |
| --- | --- |
| **iTTP-related organ injury (n.%)** |  |
| Neurological | 10 (55) |
| Cardiac | 9 (50) |
| AKI | 5 (27.8) |
| **Treatments** |  |
| Plasma exchange (1.5PM -100% FFP) (Nb. Median [IQR]) | 4 [2-5] |
| Caplacizumab (n.%) | 16 (88.9) |
| Corticosteroids (n.%) | 18 (100) |
| Rituximab (n.%) | 17 (94.4) |
| Mechanical ventilation (n.%) | 1 (5.5) |
| Vasopressors (n.%) | 1 (5.5) |
| RRT (n.%) | 0 (0) |
| **Outcome** |  |
| ICU LOS (Median [IQR]) | 6.4 [4.8-8] |
| In ICU mortality (n.%) | 1 (5.5) |
| In hospital mortality (n.%) | 1 (5.5) |

**Supplemental Table 1 abbreviations.** iTTP, immune-mediated thrombocytopenic thrombotic purpura; ICU, intensive care unit; AKI, acute kidney injury; PM, plasmatic mass; FFP, frozen fresh plasma; IQR, interquartile range; RRT, renal replacement therapy; LOS, length of stay.

**Supplemental table 2: Laser Doppler flowmetry and Ach iontophoresis raw values**

| **Microvascular laser doppler value** | (Mean ± SD) | **Baseline Perfusion index (PU)** | **Peak Perfusion index value (PU)** | **AUC** |
| --- | --- | --- | --- | --- |
| **Controls (n=34)** |  | 10.1 ± 6.3 | 67.7 ± 39.9 | 16475 ± 11738 |
| **iTTP (n=18)** | **Admission (n=16)** | 5.97 ± 4.5 | 31.9 ± 19.1 | 9627 ± 8122 |
|  | ***P* (Vs. CTR)** | 0.03 | 0.001 | 0.03 |
|  | **Post-PE1 (n=16)** | 11.38 ± 8.6 | 59.9 ± 46.7 | 16558 ± 10699 |
|  | ***P* (Vs. Admission)** | 0.027 | 0.1 | 0.07 |
|  | **Post-PE2 (n=11)** | 12.89 ± 6.9 | 92.8 ± 66.7 | 26431 ± 23181 |
|  | ***P* (Vs. Admission)** | 0.008 | 0.04 | 0.04 |

**Supplemental Table 2 abbreviations.** PU, perfusion index; CTR, controls; iTTP, immune-mediated thrombocytopenic thrombotic purpura; PE, plasma exchange; PU, perfusion index; AUC, area under curve; Ach, Acetylcholine.

**Supplemental figure 2: Biological variations during ICU stay and bivariate correlations**


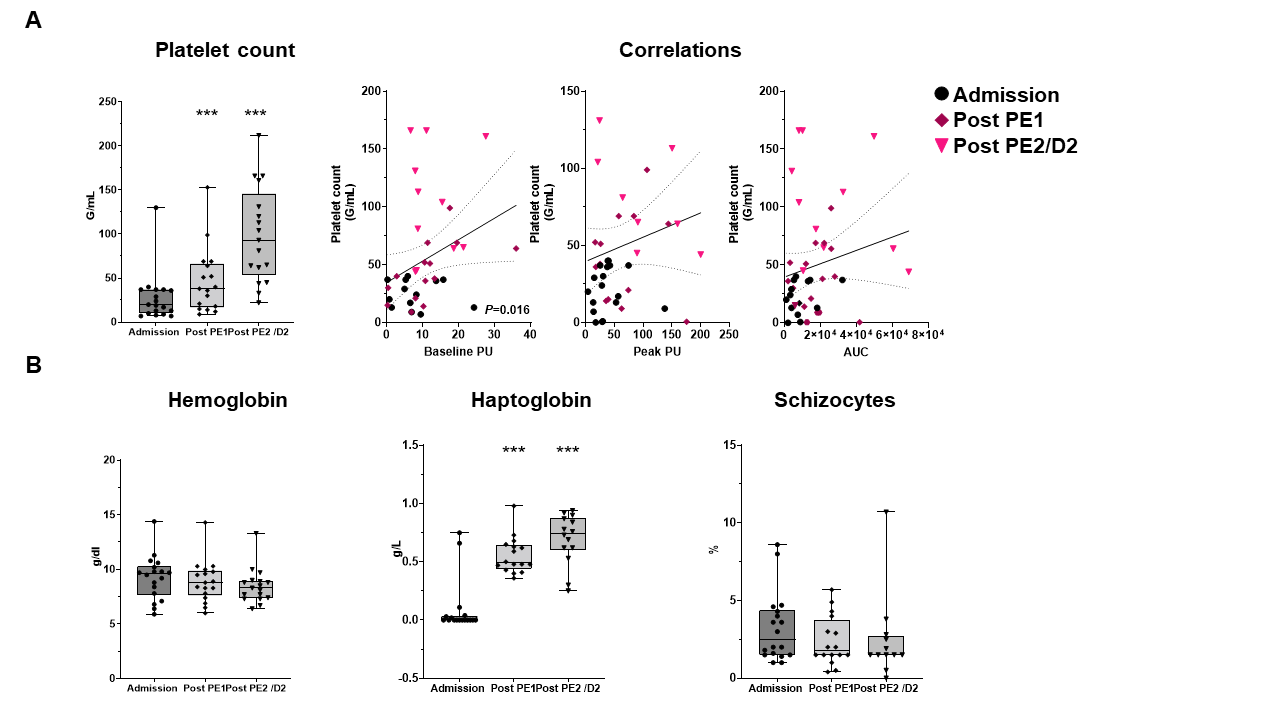


**Supplemental Figure 2 legends.** Variations of biomarkers in iTTP patients during the first days in ICU and correlation between platelet count and microvascular perfusion and reactivity flowmetry values. *** *P* < 0.0001, *versus* admission value, paired Wilcoxon signed-rank test at each time point. Abbreviations: iTTP, immune-mediated thrombocytopenic thrombotic purpura; PE, plasma exchange; LDH, lactate dehydrogenase; PU, perfusion unit; D2, day 2.

**Supplemental figure 3: Individual microvascular reactivity before and after PE.**

**Supplemental figure 3: Individual microvascular endothelium-mediated reactivity** at baseline and after PE. Red, microvascular reactivity improvement after PE; blue no improvement. Abbreviations: PE, plasma exchange; AUC, area under curve.
